# An In Vitro Comparison of Microbial Adhesion on Three Different Denture Base Materials and Its Relation to Surface Roughness

**DOI:** 10.7759/cureus.37085

**Published:** 2023-04-03

**Authors:** Nikhath Sultana, Shafath Ahmed, Vidyashree V Nandini, Jailance Lathief, Shiney Boruah

**Affiliations:** 1 Prosthodontics, SRM (Sri Ramaswamy Memorial) Kattankulathur Dental College and Hospital, Chennai, IND

**Keywords:** polyamide, candida albicans, staphylococcus aureus, surface roughness, denture base material

## Abstract

Objective: The purpose of this in vitro study is to compare and evaluate the surface roughness and microbial adhesion of *Staphylococcus aureus* and *Candida albicans* after the finishing and polishing of three different denture base materials.

Materials and Methods: A total of 84 samples of three different denture materials were used. The samples were divided into three groups: Group I (conventional poly methyl methacrylate), Group II (injection-molded polymethyl methacrylate), and Group III (injection-molded polyamide). Fourteen samples from each group were tested for surface roughness using an optical profilometer. Seven samples from each group were incubated in a suitable culture broth containing *Candida albicans* and *Staphylococcus aureus* separately for 48 hours. Microbial colony forming unit (cfu/ml^2^) was estimated in order to evaluate the microbial adhesion to the surface of the denture base materials. Confocal laser scanning microscopy was done to visualize the microorganisms.

Results: The mean surface roughness of Group I was 0.1176± 0.04 µm, Group II was 0.0669±0.02 µm, Group III was 0.1971±0.02 µm. One-way ANOVA revealed statistically significant differences in the mean surface roughness values among the three groups (p < 0.05). Tukey HSD (honestly significant difference) test confirmed the specific differences within the groups. The results of colony forming unit showed maximum adherence in Group III samples among both the species followed by Group I samples and least in Group II samples. Confocal laser scanning microscopy revealed significant differences in microbial adhesion among both *Staphylococcus aureus* and *Candida albicans* in the three groups (p <0.05). One-way multivariate ANOVA was performed to analyze the data obtained from confocal laser scanning microscopy. Microbial adhesion was least observed in Group II samples followed by Group I samples and the highest microbial adhesion was observed in Group III samples.

Conclusion: Microbial adhesion was proved to have a direct correlation with the surface roughness of denture base materials. An increase in surface roughness (Ra) increases microbial adhesion.

## Introduction

The main objective of dentistry is to improve masticatory efficiency, speech, and aesthetics by relieving pain and thereby enhancing oral health. Complete or partial edentulism is one of the most commonly encountered problems which necessitates the need to replace the missing teeth in the edentulous space. This is where the prosthodontist comes into the picture. The most commonly used material for rehabilitation is the denture base material, which evolved over time in terms of better strength, excellent fit, tissue tolerance, dimensional stability, and aesthetics.

Since 1937, polymethylmethacrylate (PMMA) has been the most widely used material for the construction of various prosthetic and orthodontic appliances [1]. Acrylic resins act as denture bases of the lost tissue as complete or removable partial dentures and various other implant-retained or tooth-supported overdentures [1-2]. Owing to its excellent mechanical and acceptable physical and aesthetic characteristics and ease of fabrication of the prosthesis, it is the most popularly used material [2]. Fabrication of prostheses is most widely done using the conventional compression molding method; nevertheless, dimensional changes have been attributed to this technique [1,3,4]. Based on several studies, the injection-molded PMMA technique has superior dimensional stability, wear strength, and water sorption properties [5-7]. Flexible polyamide was introduced with the intention to be an excellent alternative to PMMA. Valplast is a nylon thermoplastic material and it has several physical and esthetic properties.

Numerous bacteria, fungi, and viruses play a very important role in maintaining the oral flora in a healthy state. Accumulation of bacteria followed by plaque formation on the dentures can be attributed to the rough acrylic surface of the denture bases. A finely polished denture limits the accumulation of bacteria and plaque formation [8-10]. Mechanical polishing can be done by using polishing rubbers, wheels, cones, pumice slurry, and water [10-11].

So the objective of this study was to analyze the surface roughness and microbial adhesion of *Staphylococcus aureus* and *Candida albicans* on three different denture base materials in an in vitro comparison.

## Materials and methods

This in vitro study was conducted at SRM Kattankulathur Dental College and Hospital, Chennai, India. The study was approved by the Institutional Scientific and Ethical Review Board of SRM Kattankulathur Dental College and Hospital before the commencement of the study (approval number: 1796/IEC/2019). The sample size was estimated to be 28 per group, using G*Power 3.1.9.2 software with 90% power.

**Figure 1:**
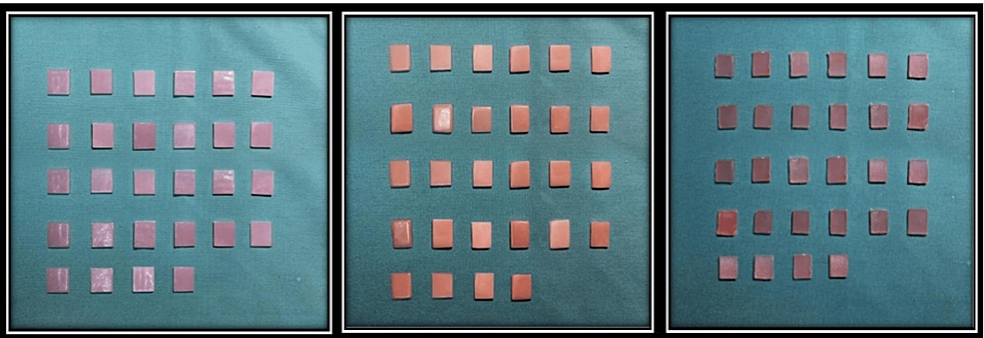
Conventional molded and injection-molded polymethyl methacrylate (A and B) Injection-molded polyamide samples (c)

 A wax pattern was fabricated using modeling wax of 3 mm in width and placed in the center of the flask using Type III dental stone. The processing was done using the conventional molded technique for Group I PMMA resin (DPI Heat Cure, Dental Products of India, Mumbai, Maharashtra, India) and injection-molded technique for Group II PMMA (SR Ivocap High Impact; Ivoclar Vivadent AG, Schaan, Liechtenstein) and Group III polyamide resin (Macro Flexi Dental Resin; Macro Dental World Pvt. Ltd., Jalandhar, Punjab, India) based on manufacturer’s instructions. After the curing cycle, bench cooling was done for 30 minutes and the acrylic sheet was retrieved. The acrylic sheets were checked for any irregularities and corrected using acrylic trimmers and sandpapers. Each acrylic sheet was cut into 28 cubes measuring 10 mm in length, 2 mm in width, and 10 mm in height using a carborundum disc (25x0.6mm) attached to the high-speed lathe at 10,000 rpm. Conventional laboratory polishing was done with a slurry of coarse pumice, water, and lathe bristle brush for 90 seconds at a rate of 1500 rpm; polished with Acrylic Polishing Kit (Shofu Inc., Kyoto, Japan) for 60 seconds at a rate of 10,000 rpm. All finishing and polishing were performed by a single operator to avoid operator variability (Figure 1).

A total of 84 samples were made. The dimensions were verified using a digital Vernier caliper. The test samples were stored at 37°C in water for 24 hrs prior to testing. The surface roughness of 14 samples from each group was evaluated using an optical profilometer (MicroXam-800, KLA Corporation, Milpitas, California, United States). The images were captured using a 1.4-megapixel camera that provided high-resolution imaging at 1360 by 1040 pixels and the images were analyzed using the software MicroXAM-800 3.0.13. Three measurements were recorded in each sample at different regions, all located in the center of the samples 1 mm away from each other. The three measurements were then averaged and the surface roughness values were recorded in micrometer (µm); the Ra parameter was used which represents the square root of the average of the square of the deviation of the scan from the mean line [12].

Fourteen samples from each group were evaluated for microbial adhesion. These 14 samples from each group were further subdivided into two groups to test the culture growth of *Staphylococcus aureus* (n=7) and *Candida albicans* (n=7). The artificial saliva was prepared using Macknight−Hane and Whitford (1992) formula and stored in glass beakers at 37^0^C [13].

**Figure 2:**
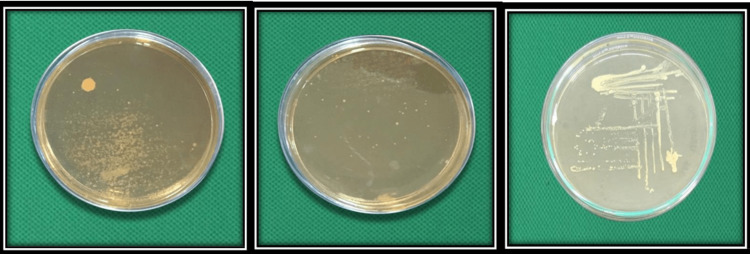
Microbial colonies obtained on culture plate, Staphylococcus aureus. Group I was categorized under 25-250cfu/ml, Group II under 25cfu/ml, and Group III under 250cfu/ml.

**Figure 3:**
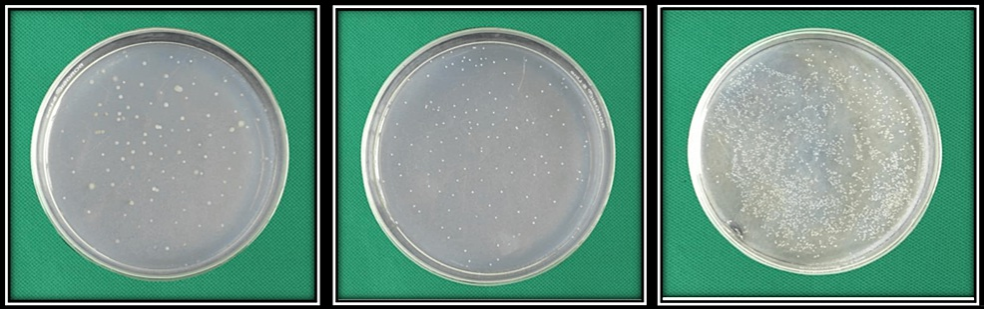
Microbial colonies obtained on culture plate, Candida albicans. Group I and Group II was categorized under 25-250cfu/ml and Group III under 250cfu/ml.

Microbial strains of *Staphylococcus aureus* - MTCC 96 and *Candida albicans* - MTCC 227 were obtained from the Microbial Type Culture Collection and Gene Bank, Chandigarh, India. The cultures were revived as per the instructions and preserved for further use. A loop full of *Staphylococcus aureus* in Luria-Bertani broth and *Candida albicans* in Potato dextrose broth (Figures 2, 3) was inoculated and incubated at 37˚C under aerobic conditions for 18 hours. Specimens were covered with artificial saliva (Preparation involves 2 gm of methyl-p-hydroxybenzoate which was dissolved in 800ml distilled water. To this solution, 0.625 gm potassium chloride, 0.059 gm magnesium chloride, 0.166 gm calcium chloride, and 0.326 gm of potassium dihydrogen phosphate were added. The second step involved the addition of 10 gm sodium carboxymethyl cellulose in 200 ml of boiling water. Once all compounds were dissolved, both solutions were mixed to obtain artificial saliva. Finally, the pH was adjusted to 6.75 by the addition of potassium hydroxide) and 5ml was kept in a petri dish and left for one hour.

Specimens were then washed using 5 ml of saline (0.89%). After washing, samples were transferred into a sterile petri dish. The cultures were centrifuged for five minutes and 5 ml of 1 x phosphate buffered solution (PBS) was added and mixed well; the absorbance was then taken at 600 nm using colorimeter (diluted the cultures 0.22). The culture in 1 x PBS was added to each specimen and a time of 15 minutes of incubation is given for pellicle formation. To this, 5% sucrose was added to each petri dish to cover all specimens completely and placed in the incubator at 37˚C for 24 hours.

The specimens were transferred into tubes containing 2 ml of PBS and centrifuged for 30 seconds to separate free bacteria. The nutrient agar and potato dextrose agar plates were spread with 100 µl of samples and checked for colony forming unit the following day [14]. Each plate was read in triplicate (three times). The final number of total microorganisms present on a surface was obtained by using the formula: Colonies/amount plated (in ml) x dilution [14]. For confocal laser scanning microscopy (CLSM), 1 mg/ml fluorescein diacetate (FDA) and 1 mg/ml ethidium bromide (ETBR) were mixed with 1 mg/ml PBS (working concentration 100 µl FDA/100 µl ETBR). To this, 5 µl of the dye was placed on the specimen and covered with a glass slide. The specimen was then viewed under CLSM.

The data obtained were statistically analyzed using IBM SPSS Statistics for Windows, Version 23.0 (Released 2015; IBM Corp., Armonk, New York, United States). The significance level was maintained at 5% (α = 0.05). One-way ANOVA and Tukey HSD (honestly significant difference) tests were used to analyze surface roughness (α ≤ 0.05). For microbial adhesion, one-way multivariate analysis and Tukey HSD tests were statically analyzed (α ≤ 0.05).

## Results

Table 1 shows the comparative analysis and standard deviation of surface roughness between the three selected denture base materials, wherein the mean values (the sum of all values divided by the total number of samples; in our case n=14) and the standard deviation (a measure of variability) is given.

**Table 1:** Comparison of surface roughness between three different denture base materials PMMA: polymethylmethacrylate

Factors	N	Mean	Std. Deviation	95% Confidence Interval for Mean	
				Lower Bound	Upper Bound
Conventional PMMA	14	.1176	.04322	.0926	.1425
Injection-molded PMMA	14	.0669	.02675	.0514	.0823
Injection-molded Polyamide	14	.1971	.01870	.1863	.2079
Total	42	.1272	.06225	.1078	.1466

The mean surface roughness value obtained for Group I (conventional molded PMMA) is 0.1176 and the corresponding standard deviation is 0.04322. For Group II injection-molded PMMA), the mean surface roughness value and standard deviation were 0.0669 and 0.02675, respectively, and for Group III (injection-molded polyamide) the mean surface roughness value and standard deviation obtained were 0.1971 and 0.01870, respectively. While an overall confidence interval of 95% was achieved, based on these results, Group II denture material is found to exhibit the least roughness in the tested area.

Among Group I samples observed for colony forming unit (cfu) for *Staphylococcus aureus* include three plates with less than 25 cfu/ml, four plates with 25-250 cfu/ml, and no plates with more than 250 cfu/ml; for *Candida albicans*, two plates with less than 25 cfu/ml, three plates with 25-250 cfu/ml, and one plate with greater than 250 cfu/ml (Table 2).

**Table 2:** Total counts of organism (cfu/ml) in Group I, conventional molded polymethylmethacrylate

Organism	Number of Samples		
	Too few to count <25cfu/ml	25-250cfu/ml	>250 TNTC cfu/ml
*Staphylococcus aureus* (n=7)	3	4	0
*Candida albicans* (n=7)	3	3	1

Among Group II samples observed for colony forming unit (cfu), for *Staphylococcus aureus* there were four plates with less than 25 cfu/ml, three plates with 25-250 cfu/ml, and none with greater than 250 cfu/ml, and for *Candida albicans*, three plates were categorized with less than 25 cfu/ml, four plates with 25-250 cfu/ml, and none with greater than 250 cfu/ml (Table 3).

**Table 3:** Total counts of organism (cfu/ml) in Group II, injection-molded polymethylmethacrylate

Organism	Number of Samples		
	Too few to count <25cfu/ml	25-250cfu/ml	>250 TNTC cfu/ml
*Staphylococcus aureus* (n=7)	4	3	0
*Candida albicans* (n=7)	3	4	0

Among Group III samples observed for colony forming unit (cfu), for *Staphylococcus aureus* two plates were categorized with less than 25 cfu/ml, four plates with 25-250 cfu/ml, and one with greater than 250 cfu/ml; for *Candida albicans*, two plates were categorized with less than 25 cfu/ml, three plates with 25-250 cfu/ml, and two plates with greater than 250 cfu/ml (Table 4).

**Table 4:** Total counts of organism (cfu/ml) in Group III, injection-molded polyamide

Organism	Number of Samples		
	Too few to count <25cfu/ml	25-250cfu/ml	>250 TNTC cfu/ml
*Staphylococcus aureus* (n=7)	2	4	1
*Candida albicans* (n=7)	2	3	2

Table 5 shows the results of multiple comparisons using Tukey HSD; it is a single-step multiple comparison procedure used to find the mean for *Staphylococcus aureus* specimens present in each group that are significantly different from each other. The mean difference Ch1-T1 variables between Group I and Group II was 8.4820; it was statistically significant (p= .000 with standard error of .82406). The mean difference between Group III and Group I was 14.7676; it was statistically significant (p=.000 with standard error of .82406). The mean difference between Group III and Group II was 23.2497; the results were found to be statistically significant (p=.000 with standard error of .82406).

**Table 5:** Tukey HSD test for comparison of Staphylococcus aureus between three different denture base materials HSD: honestly significant difference; *S. aureus*: *Staphylococcus aureus*

Dependent Variable	(I) factors	(J) factors	Mean Difference (I-J)	Std. Error	P value	95% Confidence Interval	
						Lower Bound	Upper Bound
Ch-1 T1	*S. aureus* Group I	*S. aureus *Group II	8.4820^*^	.82406	.000	6.3789	10.5852
		*S. aureus *Group III	-14.7676^*^	.82406	.000	-16.8708	-12.6645
	*S. aureus * Group II	*S. aureus* Group I	-8.4820^*^	.82406	.000	-10.5852	-6.3789
		*S. aureus *Group III	-23.2497^*^	.82406	.000	-25.3528	-21.1465
	*S. aureus* Group III	*S. aureus* Group I	14.7676^*^	.82406	.000	12.6645	16.8708
		*S. aureus *Group II	23.2497^*^	.82406	.000	21.1465	25.3528
Ch-2 T2	*S. aureus* Group I	*S. aureus *Group II	2.7270^*^	.79558	.008	.6965	4.7575
		*S. aureus* Group III	-5.8510^*^	.79558	.000	-7.8815	-3.8205
	*S. aureus* Group II	*S. aureus* Group I	-2.7270^*^	.79558	.008	-4.7575	-.6965
		*S. aureus* Group III	-8.5780^*^	.79558	.000	-10.6085	-6.5475
	*S. aureus* Group III	*S. aureus* Group I	5.8510^*^	.79558	.000	3.8205	7.8815
		*S. aureus* Group II	8.5780^*^	.79558	.000	6.5475	10.6085

The mean difference Ch2-T2 variables between Group I and Group II was 2.7270; it was statistically significant (p= .008 with standard error of .79558). The mean difference between Group III and Group I was 5.8510; it was statistically significant (p= .000 with standard error of .79558). The mean difference between Group III and Group II was 8.5780; it was statistically significant (p=.000 with standard error of .79558). An overall confidence interval of 95% was achieved, and the dependent variables (Ch1-T1 and Ch2-T2) derived were statistically significant (p = <0.05).

Table 6 shows the results of multiple comparisons using Tukey HSD for *Candida albicans* specimens present in each group that are significantly different from each other. The mean difference Ch1-T1 variables between Group I and Group II was 6.4300; it was statistically significant (p=.019 with standard error 2.11750). The mean difference between Group III and Group I was 20.6871; it was statistically significant (p= .000 with standard error 2.11750). The mean difference between Group III and Group II was 27.1171; it was statistically significant (p= .000 with standard error 2.11750).

**Table 6:** Tukey HSD test for comparison of Candida albicans between three different denture base materials HSD: honestly significant difference; *C. albicans*: *Candida albicans*

Dependent Variable	(I) factors	(J) factors	Mean Difference (I-J)	Std. Error	P value	95% Confidence Interval	
						Lower Bound	Upper Bound
Ch-1 T1	*C. albicans* Group I	*C. albicans* Group II	6.4300^*^	2.11750	.019	1.0258	11.8342
		*C. albicans* Group III	-20.6871^*^	2.11750	.000	-26.0914	-15.2829
	*C. albicans* Group II	*C. albicans* Group I	-6.4300^*^	2.11750	.019	-11.8342	-1.0258
		*C. albicans* Group III	-27.1171^*^	2.11750	.000	-32.5214	-21.7129
	*C. albicans* Group III	*C. albicans* Group I	20.6871^*^	2.11750	.000	15.2829	26.0914
		*C. albicans* Group II	27.1171^*^	2.11750	.000	21.7129	32.5214
Ch-2 T2	*C. albicans* Group I	*C. albicans* Group II	3.7243^*^	.52572	.000	2.3826	5.0660
		*C. albicans* Group III	-4.3756^*^	.52572	.000	-5.7173	-3.0339
	*C. albicans* Group II	*C. albicans* Group 1	-3.7243^*^	.52572	.000	-5.0660	-2.3826
		*C. albicans* Group 3	-8.0999^*^	.52572	.000	-9.4416	-6.7582
	*C. albicans* Group III	*C. albicans* Group 1	4.3756^*^	.52572	.000	3.0339	5.7173
		*C. albicans *Group 2	8.0999^*^	.52572	.000	6.7582	9.4416

The mean difference Ch2-T2 variables between Group I and Group II was 3.7243; it was statistically significant (p=.000 with standard error of .52572). The mean difference between Group III and Group I was 4.3756; it was statistically significant (p= .000 with standard error of .52572). The mean difference between Group III and Group II was 8.0999; it was statistically significant (p=.000 with standard error of .52572). An overall confidence interval of 95% was achieved, and the dependent variables (Ch1-T1 and Ch2-T2) derived were statistically significant (p = <0.05).

## Discussion

The process of plaque formation on the denture surface is similar to that of natural teeth. The bacterial plaque consists of various microbes mostly bacteria and fungi and this is considered to be of greater significance as this plaque can lead to a series of soft tissue changes. Symptoms such as burning mouth can develop, which can ultimately lead to denture stomatitis, chronic inflammatory hyperplasia, and candidiasis [3]. Amongst these various conditions, denture stomatitis is of more clinical significance [8]. Numerous studies in the literature have demonstrated the relationship between the plaque formed on the denture surface and denture-induced stomatitis. Studies have also evaluated the adherence of candida to various acrylic resin base materials. *Candida albicans* and oral bacteria, such as *Staphylococcus aureus*, can produce co-aggregates [15]. According to a recent paper published by Kean, *Staphylococcus aureus* and *Candida albicans* are closely connected and interact during the course of polymicrobial illness [16]. Among these microorganisms, *Candida albicans* is the most prevalent fungi (65.5%) and *Staphylococcus aureus* (34.4 %) is one of the most prevalent Gram-positive bacteria present on the prosthesis. Hence, both these organisms were selected for this in vitro study [17].

*Candida albicans* is a commensal present in the oral cavity. It usually does not cause infections in healthy individuals; however, it is an opportunistic pathogen that can cause local and systemic infections. Whereas, *Staphylococcus aureus* is a Gram-positive bacterium, which lives on the mucosa or skin surfaces. Carolus et al. (2019) explained that sometimes *Candida albicans* co-exist with commensal bacteria like *Staphylococcus aureus* and cause polymicrobial infections [17].

PMMA is the most extensively used material for the fabrication of dental prostheses by conventional heat polymerizing [18]. It is low cost, easily available, less technique sensitive, biocompatible, dimensionally stable, tasteless, odorless, less tissue irritant, non-toxic, insoluble in oral fluids, and has a good aesthetic appearance. Despite these advantages, the chief limitation of the material is polymerization shrinkage. Hence, Pryor in 1942 introduced an injection molding technique to overcome the shortcomings of conventional heat-polymerized PMMA resin [19]. He found that the continuous injection process on a closed mold compensated for shrinkage and produced a dense stronger prosthesis.

The advantage of using the injection molding technique is better dimensional stability, less polymerization shrinkage, and increased mechanical properties [15,16,20]. Aykent et al. reported the impact of the material’s physical properties on surface microbial adhesion and also the strong association between surface roughness and microbial adhesion [18]. Abuzar et al. in 2010 compared the surface roughness of a polyamide denture base material (Flexiplast) to PMMA (Vertex RS) specimens, and it was discovered that polyamide specimens created a rougher surface than PMMA, both before and after the polishing procedure [15]. These results were in accordance with the present study indicating that the variations could be attributable to the materials' physical qualities. The large difference between the two types of denture base materials is most likely owing to the unique properties of polyamide molecules, which have amide groups on the main chain and contribute to the material's hydrophilic nature.

Though there is widespread use of denture base materials in dentistry, there is no literature comparing the adhesion of both *Staphylococcus aureus* and *Candida albicans* on conventional pressure pack heat cure acrylic, injection-molded heat cure acrylic, and polyamide denture base materials. In numerous studies comparing *Candida albicans* adherence to traditional pressure pack and self-cured acrylic resins, the adherence of *Candida albicans* adherence to injection-molded acrylic materials did not reveal any significant variations [21,22].

Another finding of the present study is that amount of microbial colonization (both *Staphylococcus aureus* and *Candida albicans*) had a direct correlation with the surface roughness. There was an increase in the number of bacterial and fungal organisms in denture materials with more surface roughness.

Limitation

Limitations of this study include the following: the samples were coated with artificial saliva and ideal conditions were not stimulated. Only one investigator was involved in the study and no blinding was done. This in vitro nature may not account for changes inherent in the denture base under oral fluid conditions. Only two common microorganisms were involved in this study and also it failed to assess the depth of penetration of the microorganisms.

## Conclusions

The mean surface roughness was maximum in injection molded polyamide followed by conventional PMMA and least in injection molding PMMA denture base material. Candidal adhesion and staphylococcal adhesion were maximum in injection-molded polyamide followed by conventional PMMA and least in injection molding PMMA denture base material. The mean intensity of fluorescence in both *Staphylococcus aureus* and *Candida albicans* showed more live cells than dead cells in a confocal laser scanning electron microscope. The microbial adhesion proved a direct correlation with the surface roughness of denture base materials. Hence, an increase in surface roughness increases microbial adhesion.

The amount and tendency of a denture base material to adhere with the microorganisms should also be considered before restoring the prosthesis because these prostheses will be in direct contact with the microflora of the mouth and they might lead to biofilm formation on the surfaces and can cause infections locally or systemically. This is critical in providing a prosthesis with the least microbial adherence. So, the search for new materials with superior properties and the least microbial adhesion should continue. Further in vivo studies need to be conducted to evaluate the microbial adhesion on various other materials and the depth of penetration of the microorganisms also should be assessed.
